# Modeling human Coenzyme A synthase mutation in yeast reveals altered mitochondrial function, lipid content and iron metabolism

**DOI:** 10.15698/mic2015.04.196

**Published:** 2015-04-06

**Authors:** Camilla Ceccatelli Berti, Cristina Dallabona, Mirca Lazzaretti, Sabrina Dusi, Elena Tosi, Valeria Tiranti, Paola Goffrini

**Affiliations:** 1 Department of Life Sciences, University of Parma, Parma, Italy.; 2 Unit of Molecular Neurogenetics - Pierfranco and Luisa Mariani Center for the study of Mitochondrial Disorders in Children, IRCCS Foundation Neurological Institute “C. Besta”, Milan, Italy.

**Keywords:** Saccharomyces cerevisiae, yeast model, Coenzyme A, NBIA, COASY, mitochondria, iron accumulation, lipid content

## Abstract

Mutations in nuclear genes associated with defective coenzyme A biosynthesis have been identified as responsible for some forms of neurodegeneration with brain iron accumulation (NBIA), namely PKAN and CoPAN. PKAN are defined by mutations in *PANK2*, encoding the pantothenate kinase 2 enzyme, that account for about 50% of cases of NBIA, whereas mutations in CoA synthase *COASY* have been recently reported as the second inborn error of CoA synthesis leading to CoPAN. As reported previously, yeast cells expressing the pathogenic mutation exhibited a temperature-sensitive growth defect in the absence of pantothenate and a reduced CoA content. Additional characterization revealed decreased oxygen consumption, reduced activities of mitochondrial respiratory complexes, higher iron content, increased sensitivity to oxidative stress and reduced amount of lipid droplets, thus partially recapitulating the phenotypes found in patients and establishing yeast as a potential model to clarify the pathogenesis underlying PKAN and CoPAN diseases.

## INTRODUCTION

In all living cells Coenzyme A (CoA) is the major carrier of acetyl and acyl groups playing a central role in basic cellular functions such as lipids metabolism, Krebs cycle and aminoacid biosynthesis. CoA biosynthesis proceeds through a highly conserved pathway, involving five enzymatic steps: pantothenic acid (vitamin B5) phosphorylation, cysteine conjugation, decarboxylation, conjugation to an adenosyl group and phosphorylation.

Whereas in mammals the last two steps are catalyzed by Coenzyme A synthase (COASY), a mitochondrial bifunctional enzyme possessing both 4’-phospho-pantetheine adenylyltransferase (PPAT) and dephospho-CoA kinase (DPCK) activities 1,2, in other organisms, such as yeast, PPAT and DPCK activities reside in two different enzymes, Cab4 and Cab5, the products of the essential genes *CAB4 *and* CAB5 *3 whose compartmentalization is not well understood.

Recently, it has been reported that dysfunctions of CoA biosynthetic pathway may play a role in the pathogenesis of neurodegeneration with brain iron accumulation (NBIA), a wide spectrum of clinically and genetically heterogeneous diseases characterized by progressive neurodegeneration and high iron content in specific brain region 4,5,6.

This concept is supported by the fact that mutations in *PANK2,* encoding the first enzyme in the CoA synthesis, approximately account for 50% of NBIA cases, classified as PKAN (Pantothenate Kinase Associated Neurodegeneration) 7,8. Moreover *COASY* gene has been identified as a novel NBIA-associated gene and these NBIA cases have been termed CoPAN (COASY Protein-Associated Neurodegeneration)^9^.

Neurodegenerative diseases are often characterized by mitochondrial dysfunctions, altered lipid metabolism and iron accumulation 10,11,12 and several evidences linking PKAN and CoPAN to these metabolic alterations have been reported 8,13,14,15,16.

The development of cellular and animal models is crucial for advancing our understanding of the pathophysiology of these diseases. In the last decade, the yeast *Saccharomyces cerevisiae *has been used as *in vivo* model system to gain insights into the molecular basis of mitochondrial pathologies and neurodegenerative disorders 17,18. Despite their simplicity, yeast cells possess most of the basic cellular machinery including pathways required for energy metabolism and protein homeostasis. Moreover, many of the genes and biological systems that function in yeast iron homeostasis are conserved throughout eukaryotes 19.

To investigate if defective CoA metabolism could underlie a more general disequilibrium of lipid metabolism and mitochondrial dysfunctions and its relationship with brain iron accumulation, we have performed phenotypic and biochemical investigation in a recently developed yeast model expressing the pathogenic missense mutation *COASY*^R499C^ found in NBIA patients 9.

The results obtained in this study showed that yeast mutant defective in CoA biosynthesis recapitulates the most important phenotypes found in patients and validated this system to model CoPAN in order to help elucidating important cellular and biochemical aspects of mitochondrial, lipid and iron homeostasis underpinning this disease.

## RESULTS

### Cellular localization of yeast Cab5 protein

**Figure 1:**
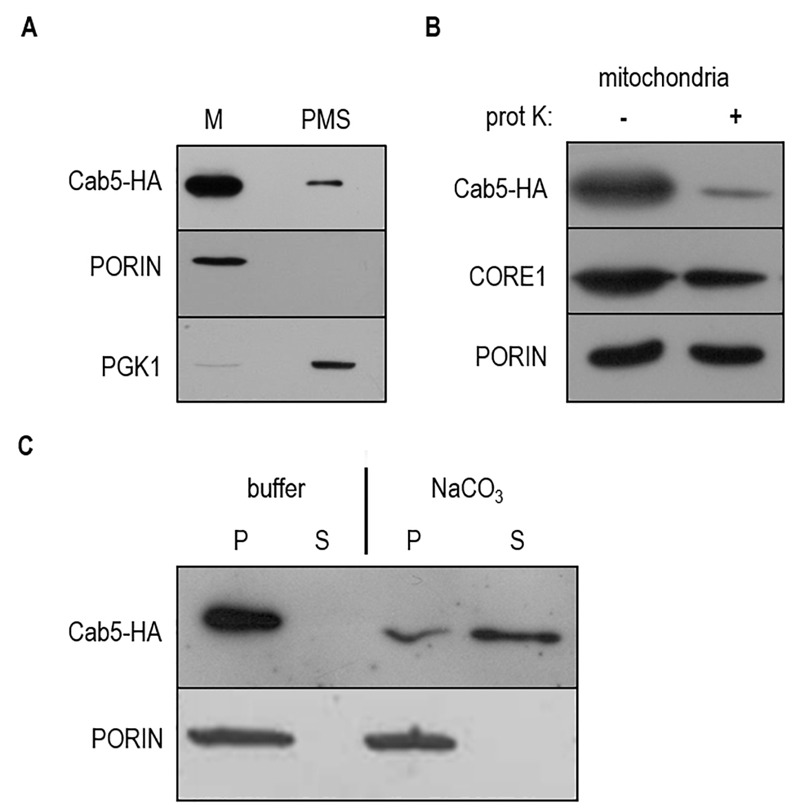
FIGURE 1: Localization of Cab5p. ** (A)** Equal amounts (20 mg) of the mitochondrial fraction (M) and post mitochondrial fraction (PMS) were resolved by SDS-PAGE and analyzed by immunoblotting with HA, PGK1 (cytosolic marker), PORIN (mitochondrial outer membrane marker) antibodies. ** (B)** Mitochondria were treated for 60 min at 4°C with proteinase K (prot K) (1 mg/ml). The filter was incubated with anti-HA, anti-CORE1, and anti-PORIN antibodies. Core1 was used as an inner membrane protein control. ** (C)** 150 µg of mitochondrial proteins were treated with TEGN buffer or TEGN and 0.1M NaCO_3. _The insoluble pellet (P) and supernatant (S) fractions were resolved by SDS-PAGE and analyzed by immunoblotting with HA and PORIN antibodies.

Proteomics studies 20 and *in silico* analysis using the PSORT and MITOPROT programs 21,22, which allows the prediction of protein localization, suggest for Cab5 a mitochondrial localization. Moreover human CoA synthase is a mitochondrial enzyme and the human gene is able to complement the *cab5*Δ mutation. To confirm experimentally the mitochondrial localization of Cab5p, a carboxyl-terminal fusion of HA epitope to Cab5 was constructed. The *cab5*Δ lethal phenotype was rescued by the re-expression of the tagged wild type allele, indicating that the addition of HA did not disrupt targeting and function of the Cab5 protein. Equivalent amounts of mitochondrial pellet (M) and supernatant (PMS) fractions from cells expressing HA tagged Cab5 (Cab5-HA) were subjected to SDS-PAGE and Western blotting to identify the indicated protein. The great majority of Cab5-HA co-fractionated with the mitochondrial membrane protein porin, while only a small amount remained in the supernatant with the soluble cytoplasmic protein phosphoglycerate kinase (PGK), indicating that Cab5 behaves as a mitochondrial associated protein (Fig. 1A). We further investigated the mitochondrial localization of Cab5p by performing a protease protection assay of intact mitochondria. Cab5-HA exhibited a significant increase in proteinase K sensitivity treatment in respect to both porin, which is only partially exposed on the surface, and to the inner membrane protein Core1 (Fig. 1B). The mitochondria were then treated with 0.1 M Na_2_CO_3_, pH 11, and supernatant and pellet fractions were generated by centrifugation. As depicted in Fig. 1C the amount of Cab5 associated with mitochondria was significantly reduced but the amount of porin was not altered. Taken together these results suggest that Cab5 is an extrinsic outer membrane protein.

### Characterization of mitochondrial functions

We have previously demonstrated by HPLC analysis that in the strain expressing the human *COASY*^R499C ^or the yeast *cab5*^R146C ^mutant versions the level of CoA in mitochondria was reduced by 40% compared to wild-type 9. Given that defective CoA biosynthesis could lead to a variety of metabolic defects we looked for evidence of mitochondrial dysfunction.

**Figure 2:**
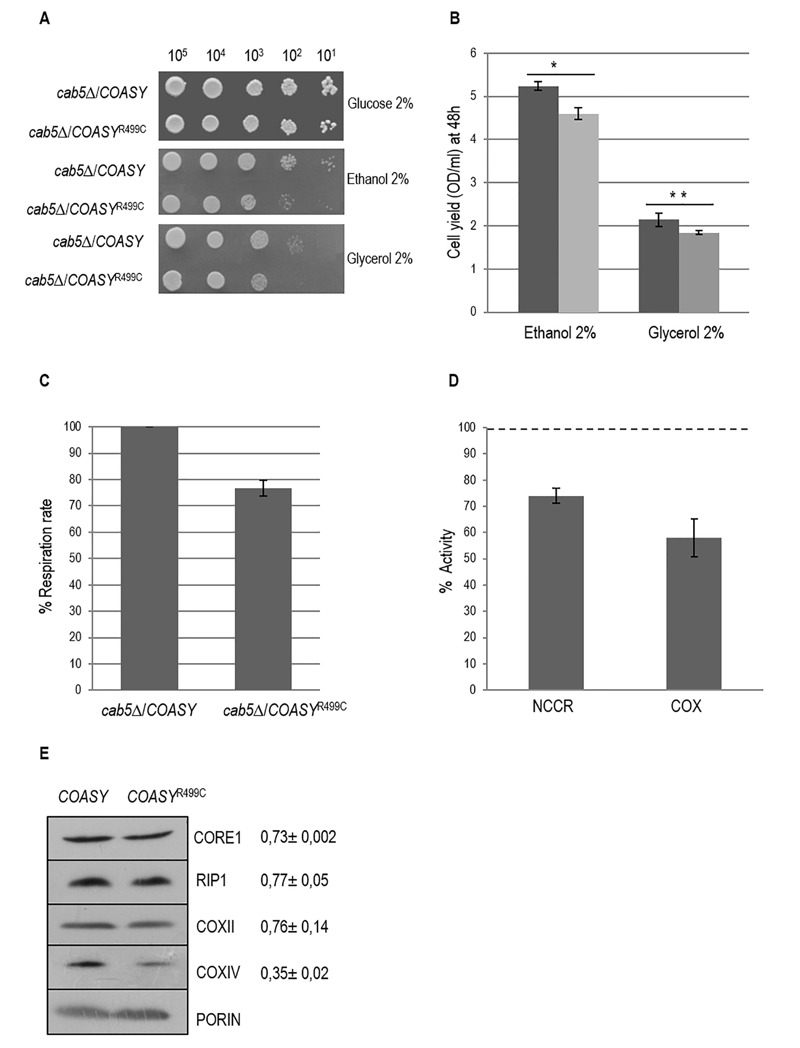
FIGURE 2: Characterization of mitochondrial functions. ** (A)** Oxidative growth phenotype. The strain W303-1B *cab5*Δ was transformed with a pYEX-BX plasmid carrying the wild-type *COASY* or the mutant allele* COASY*^R499C^. Equal amounts of serial dilutions of cells from exponentially grown cultures (10^5^, 10^4^, 10^3^, 10^2^, 10^1^) were spotted onto mineral medium (40) plus 2% glucose, 2% ethanol or 2% glycerol without pantothenate. The growth was scored after 5 days of incubation at 28°C. ** (B)** Cell yield. Cell yield was calculated by growing cells on liquid medium containing ethanol or glycerol and measuring the optical density at 600 nm after 48h of growth (*COASY* black columns and *COASY*^R499C^ grey columns). Values are mean of three independent experiments. * P < 0.05; **P < 0.01 (unpaired two-tailed t-test). ** (C)** Oxygen consumption rates. Respiration was measured in cells grown in mineral medium (40) plus 0.2% glucose and 2% galactose without pantothenate at 28°C. The values observed for the *COASY* mutant strain are reported as percentage of the respiration obtained in cells expressing the wild-type *COASY* gene. ** (D)** NADH-cytochrome *c* oxidoreductase (NCCR) and cytochrome *c *oxidase (COX) activities were measured in mitochondria extracted from cells grown exponentially at 28°C in mineral medium (40) plus 0.2% glucose and 2% galactose without pantothenate. The values of the *COASY *mutant are expressed as percentage of the activities obtained in the wild type strain. ** (E)** Steady state level of cIII and cIV subunits in cells carrying the wild-type *COASY* and the mutant allele. The filter was incubated with specific antibodies against Core1, Rip1, CoxII, CoxIV and Porin. The signals were normalized according to the control signal (porin) and taken as 1.00 the signal of the *cab5*∆/*COASY* (wild-type) strain.

In order to reveal a possible respiratory growth defect, serial dilutions of the strains were spotted on mineral medium without pantothenate supplemented with either ethanol or glycerol, at 28°C. As shown in Fig. 2A the OXPHOS growth of the *cab5*Δ/*COASY*^R499C ^mutant was partially affected compared to *COASY* wild type expressing strain. To confirm the growth delay we determined the cell

yield for each yeast strain grown on ethanol or glycerol. We observed that the OXPHOS growth of the mutant strain was 20% lower as compared to wild type (Fig. 2B).

To further analyze the respiratory deficiency, oxygen consumption and activity of respiratory complexes were measured. Accordingly to the OXPHOS growth phenotype the oxygen consumption rate of the *cab5*Δ/*COASY*^R499C ^was 25% less than that of *cab5*Δ/*COASY* (Fig. 2C). Likewise, the NADH-cytochrome *c* oxidoreductase (NCCR) and cytochrome *c* oxidase (COX) activities were reduced in the mutant strain respectively to 26% and 42% as compared to wild type (Fig. 2D). Accordingly, the steady state levels of complex III and IV subunits are decreased (Fig. 2E). Altogether these results indicate a mitochondrial dysfunction associated to the reduced CoA level.

### Mutation in CoA synthase determines an increase of iron content and increased sensitivity to oxidative stress

NBIA disorders, PKAN and CoPAN included, are characterized by iron deposition in the brain but the mechanisms leading to iron overload and its pathophysiological role remain unclear. Since in yeast excessive iron accumulation in the mitochondria led to an increased sensitivity to this ion 23,24, we first evaluated the inhibition of cellular growth in the *COASY*^R499C^ mutant strain by the addition of FeSO_4_ to the medium.

**Figure 3:**
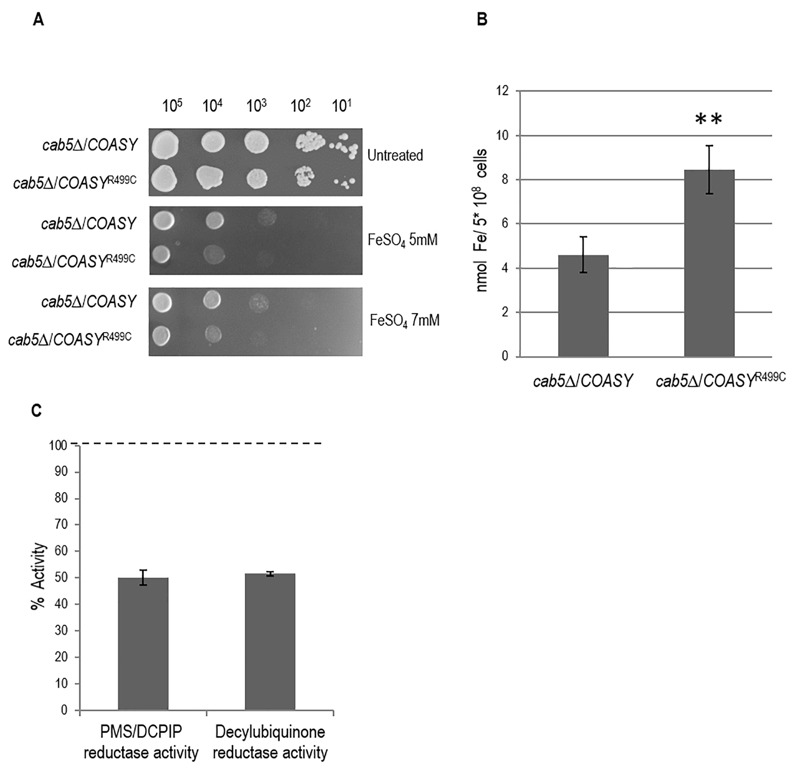
FIGURE 3: Measurement of iron content. ** (A)** Iron sensitivity. Equal amounts of serial dilutions of cells from exponentially grown cultures (10^5^, 10^4^, 10^3^, 10^2^, 10^1^) were spotted onto 40 medium without pantothenate supplemented with 5 mM and 7 mM FeS0_4_. The growth was scored after 5 days of incubation at 28°C. ** (B)** Iron level. Cellular iron levels was quantified in cells grown up to early stationary phase in YNB glucose (0,6%) medium. **P < 0.01 (unpaired two-tailed t-test). ** (C)** PMS/DCPIP (phenazyne methosulfate/dichlorophenolindophenol) reductase and decylubiquinone reductase activities were measured in mitochondria extracted from cells grown exponentially at 28°C in mineral medium (40) plus 0.2% glucose and 2% galactose without pantothenate. The values of the COASY mutant are expressed as percentage of the activities obtained in the wild type strain.

As depicted in Fig. 3A, the mutant strain showed a clear growth defect when compared to wild type strain, indirectly indicating a higher iron content.

We then performed a quantitative determination of cellular iron level by a colorimetric assay that relies on the formation of colored iron complexes with BPS after nitric acid digestion of yeast cells and gives results comparable with those with ICP-mass spectrometry 25,26. The results obtained showed a two-fold increase in iron content in the *COASY*^R499C^ mutant respect to the parental strain (Fig. 3B).

We then investigated whether the biosynthesis of the Fe-S cluster, a marker of mitochondrial functionality linked to iron metabolism, was affected by COASY deficiency. We determined the activity of succinate dehydrogenase (SDH), a mitochondrial Fe-S cluster enzyme. As shown in Fig. 3C, SDH activity was decreased by about 50%, in the mutant as compared to wild-type strain.

**Figure 4:**
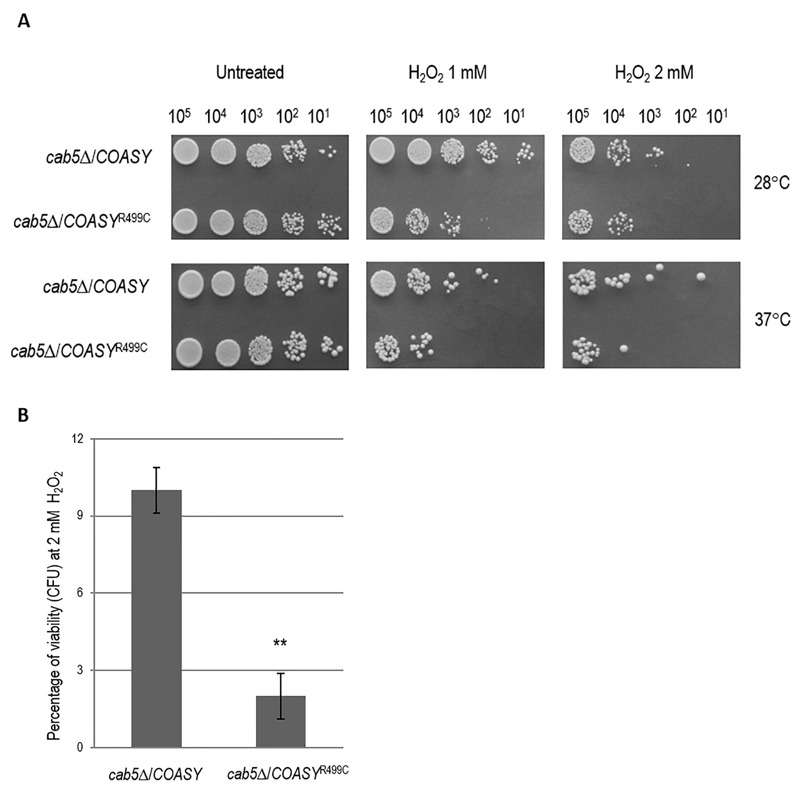
FIGURE 4: Sensitivity to H_2_O_2_. ** (A)** The cells carrying the wild-type *COASY* or mutant allele were grown up to exponential phase and incubated for 4h at 28°C or 37°C with the addition of 1 mM and 2 mM H_2_O_2_. After the treatment, equal amounts of serial dilutions of cells (10^5^, 10^4^, 10^3^, 10^2^, 10^1^) were spotted onto YP media plus 2% glucose. The growth was scored after 2 days of incubation at 28°C or 37°C. ** (B)** Viability of wild-type and mutant strains was measured by C.F.U counting after exposure of cell to 2 mM H_2_O_2_ for 4h.**P < 0.01 (unpaired two-tailed t-test).

It is known that an excess of iron causes an altered oxidative status 24,27,28, another key feature of disease associated to CoA deficiency 14,29,30, which may be reflected in hypersensitivity to oxidative stress-induced cell death. To test this hypothesis *COASY*^R499C^ mutant and control strain were exposed to H_2_O_2_ and cell viability was determined by both spot assay analysis (Fig. 4A) and by counting the formation of colonies (Fig 4B). At the highest H_2_O_2 _concentration tested (2 mM) wild type cells showed a viability of 10%, while mutant cells showed a viability of 2% (Fig. 4B) demonstrating that a COASY defect leads to oxidative stress susceptibility.

### Evaluation of lipid droplets content

**Figure 5:**
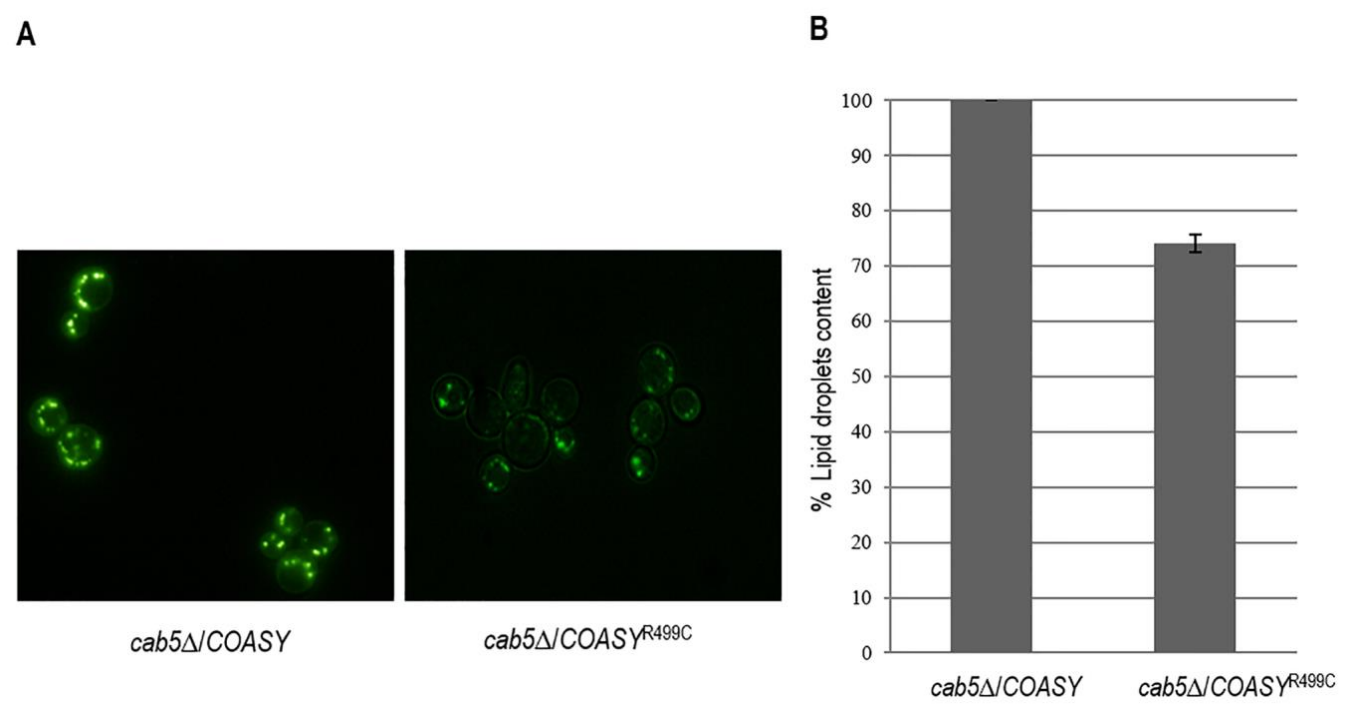
FIGURE 5: Evaluation of lipid droplets content. The intracellular lipid droplets content has been detected by fluorescence microscopy ** (A)**and fluorimetric analysis ** (B)** after incubation of wild type and mutant cells with the fluorescent lipophilic dye Nile Red (4 μg/ml). The values corresponding to mutant *COASY* are expressed as percentage of the content obtained in the wild type strain.

Acetyl-CoA is necessary for the production of neutral lipids, which serve as power reserve for the cell and are stored in lipid droplets. Since CoA is involved in the biosynthesis of fatty acids and having demonstrated that the mutant *cab5*Δ/*COASY*^R499C^ shows a 40% reduction of coenzyme A, the content of intracellular lipid droplets in the mutant compared to the wild type was evaluated by fluorescence microscopy and fluorimetric analysis after incubation of the cells with the fluorescent lipophilic dye Nile Red 31. As shown in Fig. 5A the content of lipid droplets is decreased in the mutant expressing the *COASY*^R499C^. In order to better quantify this reduction, the fluorescence of cells stained with Nile Red was measured using a fluorescence spectrometer. The measures, performed in triplicate, highlighted a reduction of lipid droplets of 25% in mutant strain compared to wild-type (Fig. 5B).

## DISCUSSION

In all living organisms Coenzyme A (CoA) is an essential cofactor in cellular metabolism. CoA biosynthesis follows a highly conserved pathway, involving five enzymatic steps, which utilize pantothenate (vitamin B5), ATP, and cysteine. Mutations in nuclear genes directly involved in CoA biosynthetic pathway have been identified as responsible for some forms of NBIA, namely PKAN and CoPAN. PKAN is caused by mutations in PANK2, encoding the pantothenate kinase 2 enzyme, that account for about 50% of NBIA cases, whereas mutations in CoA synthase COASY have been recently reported as the second inborn error of CoA synthesis leading to CoPAN 9. In PKAN and CoPAN brain iron accumulation is dramatic but its link with defective CoA synthesis is unknown.

Moreover, many neurodegenerative diseases, PKAN and CoPAN included, are characterized by mitochondrial dysfunctions, oxidative stress, altered lipid metabolism but again the complex relationships linking these factors in the context of disease conditions remain to be elucidated.

Previous attempts to understand the mechanism of PKAN using animal models have met with limited success. A mouse model of PKAN exhibits azoospermia but lacks any neurological phenotype 32. A *Drosophila* model of PKAN does have a brain phenotype, but this involves the formation of vacuoles, not iron accumulation 33. The identification and generation of other cellular model may allow a deeper characterization of *COASY *and *PANK2* disease gene products, and the investigation of their pathophysiology *in vivo*. With this aim we developed and characterized a yeast model for CoPAN disease.

Although in yeast, differently from mammalian cells, the last two steps of CoA biosynthesis are catalyzed by two separate enzymes, namely the products of the essential genes *CAB4* and *CAB5*, we have demonstrated that the lethality associated to deletion in* CAB5* could be complemented by human *COASY*. This allowed us to study the human Arg499Cys substitution in yeast and to support the pathogenic role of this mutation associated to a reduced level of CoA 9.

The evaluation of the metabolic consequences of coenzyme A deficiency in yeast revealed mitochondrial dysfunctions; OXPHOS growth was affected and respiration rate significantly decreased. Accordingly, the activity of respiratory chain complexes and steady state levels of mitochondrial respiratory chain subunits were reduced. We also demonstrated that the growth of the mutant strain is not only strongly inhibited in the presence of iron but that the mutant strain showed iron accumulation. This result is consistent with the patient phenotype, with iron overload being a typical sign of PKAN and CoPAN. We have also found that *cab5*Δ/*COASY*^R499C^ mutant was more sensitive to the ROS-inducing agent H_2_O_2 _indicating an increased oxidative stress that may contribute to the pathogenesis of these diseases. Accordingly, the activity of SDH, a marker of mitochondrial functionality linked to iron metabolism, was reduced in the *COASY* mutant.

Since Acetyl-CoA, one of the most important derivatives of CoA, is also required for the synthesis of fatty acids, we investigated the impact of reduced CoA level by measuring the lipid droplets content in mutant cells by fluorimetric analysis of Nile Red stained cells. Interestingly enough, lipid droplets content was 25% lower in mutant strain as compared to wild-type. The same altered lipid metabolism was also observed in mutant strain for phosphopantothenoylcysteine synthetase (PPCS), another essential enzyme in CoA biosynthetic pathway 34. These results are in agreement with the hypothesis that low CoA perturbs lipid homeostasis; lipid deregulation was also observed in *Drosophila* CoA mutants and from global metabolic profiling studies in patient-derived fibroblasts 8,35. The transcriptional analysis of key genes involved in lipid metabolism would help in elucidating the role of lipid metabolism in the pathology.

Altogether these results established yeast as an appropriate model to study the molecular mechanisms involved in CoA metabolism, and to understand the connection between iron management, mitochondrial function and lipid metabolism in neurodegeneration.

Several pathological phenotypes have been identified in the mutant *COASY* yeast strain thus representing ideal readouts for high throughput screening of chemical libraries as described by Couplan *et al.* 36. This could allow the identification of new molecules, the first step to set up future therapeutic experimental approaches.

## MATERIALS AND METHODS

### Yeast strains, media and vectors 

Yeast strains used in this study were W303-1B *cab5*Δ (MATα; *cab5*::*KanMx4 ade2-1 leu2-3,112 ura3-1 his3-22*,*15 trp1-1 can1-100*) carrying pYEX-BX-COASY or pYEX-BX COASY^R499C^ plasmid 9. For localization studies we used the strain BY4741 (MAT*a*; *his3*∆*1* *leu2*∆*0* *met15*∆*0* *ura3*∆*0*) transformed with pFL38-Cab5-HA plasmid.

Cells were cultured in mineral medium (40) supplemented with appropriate amino acids and bases for auxotrophy as previously described 37. To obtain medium lacking pantothenate (40-Pan) a mixture of vitamins without pantothenate was prepared. Yeast cells were transformed by the standard lithium acetate method 38 and cultured in YNB synthetic defined media (For-MediumTM, UK) supplemented with 1 g/l of drop-out powder 39 containing all amino acids and bases, except those required for plasmid selection. Various carbon sources (Carlo Erba Reagents, Italy) were added at the indicated concentration. Media were solidified with 20 g/l agar (For-Medium™). Strains were incubated at 28 or 37°C as indicated.

Plasmid pFL38-Cab5-HA was obtained by PCR overlap technique 40. In the ﬁrst set of PCR reactions, the *CAB5* region was obtained using the forward primer CAB5Fw-GGGGGGATCCCCATTGCTTAGAATGGGCGG and the following reverse tag primer CAB5HARv ATCAACCTTATAC**AGCGTAATCT-GGAACATCGTATGGGTACGCTGA**AGACTTTTTATTTTG where hemagglutinin (HA) tag sequence is indicated in bold. The second *CAB5* region was obtained using the forward tag primers CAB5HATERFw complementary to CAB5HARv , and the reverse primer CAB5Rv-CCGCGGTACCGAGAACCCATAGAATT-CGAC. The ﬁnal product was obtained using the overlapping PCR fragments as template with CAB5Fw and CAB5Rv as external primers. The product was then digested with *BamH*I and *Kpn*I and cloned in *BamH*I/*Kpn*I digested pFL38 centromeric plasmid 41.

### Respiration measurement, biochemical assays and immunoblot analysis of respiratory chain subunits.

Respiratory activity measurement was performed on whole cells at 30°C using a Clark-type oxygen electrode (Oxygraph System, Hansatech Instruments, England) with 1 ml of air-saturated respiration buffer (0.1 M phthalate-KOH, pH 5.0). The reaction started by addition of 20 mg of wet-weight cells 42.

Complex II (SDH), NADH-cytochrome *c* oxidoreductase (NCCR) and complex IV specific activities were measured spectrophotometrically as previously described 42,43,44,45 on a mitochondrial enriched fraction prepared according to Soto *et al*. 46. Protein concentration was determined by the method of Bradford using the BioRad protein assay following manufacturer’s instructions 47. For SDS-PAGE, 20 μg of mitochondrial proteins were separated on 12% polyacrylamide gels and electroblotted onto a nitrocellulose membrane. The subunits of mitochondrial respiratory complexes were immunovisualized by specific antibodies. The sources of primary antibodies are indicated: anti-Core1 (a kind gift from Prof. Antoni Barrientos), anti-Rip1 (a kind gift from Prof. Alexander Tzagoloff), anti CoxIIp (Abcam Mitoscience), anti-CoxIV (Abcam Mitoscience) and anti-Porin (Abcam Mitoscience). Quantification of protein bands was performed using Multi Analyst software. The signals were normalized according to the control signal (α-Porin) and the signal of the *cab5*∆/COASY (wild-type) strain was set as 1.00.

### Intact mitochondria isolation, subcellular localization experiments and membrane association

Intact mitochondria were isolated from BY4741 strain transformed with a plasmid expressing Cab5-HA under the native *CAB5* promoter after cell wall removal by Zymoliase20T digestion (Nacalai Tesque) and cell disruption with a glass-teflon potter homogenizer 48. Whole cell extract was centrifuged at 12,000 g for 30 min to yield the mitochondrial pellet (M) and post-mitochondria supernatant (PMS). These fractions were analyzed by immunoblotting with the indicated antibodies (Porin: mitochondrial marker; PGK cytoplasmic marker (Abcam Mitoscience)). The Cab5 protein was immunovisualized using an anti-HA (Roche) specific antibody. Proteinase K protection assay for sub-mitochondrial localization study was performed as previously described 48,49. Briefly, 200 μg of mitochondrial proteins were kept in 20 mM HEPES pH 7.4, 0,6 M sorbitol in the presence or absence of proteinase K (1 mg/ml) for 60 minutes on ice. 0,1 M PMSF (phenylmethylsulfonyl ﬂuoride) was added to stop the reaction. The protein pellets were washed once with 20 mM HEPES pH 7.4 plus 0,6 M sorbitol, and suspended in SDS-PAGE sample buffer.

A modified version of the membrane association experiments of Trott and Morano 50 was utilized to determine the resistance of Cab5p to sodium carbonate (pH 11.5) treatment. Equal amounts (150 μg) of the mitochondrial fraction was resuspended in TEGN (20 mM Tris-HCl [pH 7.9], 0.5 mM EDTA, 10% glycerol, 50 mM NaCl) or TEGN and with 0.1 M NaCO_3_ for 30 min on ice. The samples were subsequently centrifuged at 17,000 g at 4°C to obtain soluble and membrane fractions. The fractions obtained in all type of extraction were separated by SDS-PAGE and probed with anti-HA and anti-PORIN antibodies.

### Measurement of iron content 

The iron content was determined by a colorimetric assay, essentially as described before 25,51. 5x10^8 ^cells were washed twice with H_2_O, resuspended in 0.5 ml of 3% nitric acid and incubated over night at 95°C. After incubation, samples were centrifuged at 12,000 rpm for 5 min and the supernatant (400 µl) was mixed with 160 µl of 38 mg sodium L-ascorbate ml^- 1 ^(SIGMA), 320 µl of 1.7 mg BPS ml^-1^ (ACROS ORGANICS), and 126 µl of ammonium acetate (SIGMA) (saturated solution diluted 1:3). Non-specific absorbance was measured at 680 nm and subtracted from the specific absorbance of the iron-BPS complex (535 nm). Iron was quantified by reference to a standard curve using iron sulfate performed as in Tamarit *et al*. 25.

### H_2_O_2_ sensitivity

To determine the sensitivity to oxygen peroxide, cells growing exponentially were exposed to 1 and 2 mM H_2_O_2_ at 28°C or 37°C for 4 h. Cell viability was determined by spotting equal amounts of serial dilutions of cells (10^5^, 10^4^, 10^3^, 10^2^, 10^1^) onto YP plates (1% yeast extract, 2% peptone ForMedium^TM^) supplemented with 2% glucose (YPD). Plates were incubated at 28°C or 37°C for two days. To better quantify H_2_O_2_ sensitivity cell survival was determined by counting the formation of colonies after the treatment.

### Lipid droplets content: fluorescence microscopy and fluorimetric analysis 

Intracellular lipid droplets were detected using the fluorescent lipophilic dye Nile Red (9-diethylamino-5*H*-benzo[α]phenoxazine-5-one 3 SIGMA-ALDRICH) by fluorescence microscopy and fluorimetric analysis 31,52,53. Wild type and *cab5*∆/*COASY^R499C^* strains were grown to mid-log phase in mineral medium (40) containing Yeast Extract (1,5 g/L). To 250 μl of the cultures, adjusted to 1 OD, 10 μl of the stock solution of Nile red [100 μg/ml] were added in order to obtain a final concentration of 4 µg/ml of dye. Fluorescence of the stained cells were obtained with a Leica DM2000 microscope using x 100 magnification and captured using a Leica DFC310FX digital camera with Leica’s Imaging Software (Leica Application Suite-LASAF 3.7.0, Leica Microsystem).

To quantify the fluorescence we used the fluorescence spectrometer Tecan SPECTRA Fluor Plus using the software XFLUOR4 V4.51 (excitation at 535 nm and emission at 595 nm). Aliquots of 100 μl of cells stained with Nile red were transferred into 96-well microplates in 4 replicates. For each strain a negative control was performed in which the dye was omitted in order to exclude a possible auto fluorescence of samples. The evaluation of the fluorescence was repeated at 5-minute intervals in a time interval of 20 minutes 53.

## References

[1] Zhivoloup A, Nemazanyy I, Babich A, Panasyuk G, Pobigailo N, Vudmaska M, Naidenov V, Kukharenko O, Palchevskii S, Savinska L, Ovcharenko G, Vardier F, Valovka T, Fenton T, Rebholz H, Wang M, Sheperd P, Matsuka G, Filonenko V, Gout IT (2002). Molecular cloning of CoA Synthase. The missing link in CoA biosynthesis.. The Journal Of Biological Chemistry.

[2] Zhyvoloup A, Nemazanyy I, Panasyuk G, Valovka T, Fenton T, Rebholz H, Wang ML, Foxon R, Lyzogubov V, Usenko V, Kyyamova R, Gorbenko O, Matsuka G, Filonenko V, Gout IT (2003). Subcellular localization and regulation of coenzyme A synthase.. J Biol Chem.

[3] Olzhausen J, Schübbe S, Schüller H (2009). Genetic analysis of coenzyme A biosynthesis in the yeast Saccharomyces cerevisiae: identification of a conditional mutation in the pantothenate kinase gene CAB1.. Current Genetics.

[4] Gregory A, Hayflick SJ (2005). Neurodegeneration with brain iron accumulation.. Folia Neuropatho.

[5] Brunetti D, Dusi S, Morbin M, Uggetti A, Moda F, D’Amato I, Giordano C, d’Amati G, Cozzi A, Levi S, Hayflick S, Tiranti V (2012). Pantothenate kinase-associated neurodegeneration: altered mitochondria membrane potential and defective respiration in Pank2 knock-out mouse model.. Human Molecular Genetics.

[6] Hayflick SJ (2014). Defective pantothenate metabolism and neurodegeneration.. Biochem Soc Trans.

[7] Hartig MB, Hörtnagel K, Garavaglia B, Zorzi G, Kmiec T, Klopstock T, Rostasy K, Svetel M, Kostic VS, Schuelke M, Botz E, Weindl A, Novakovic I, Nardocci N, Prokisch H, Meitinger T (2006). Genotypic and phenotypic spectrum of PANK2 mutations in patients with neurodegeneration with brain iron accumulation.. Annals Of Neurology.

[8] Leoni V, Strittmatter L, Zorzi G, Zibordi F, Dusi S, Garavaglia B, Venco P, Caccia C, Souza AL, Deik A, Clish CB, Rimoldi M, Ciusani E, Bertini E, Nardocci N, Mooth VK, Tiranti V (2012). Metabolic consequences of mitochondrial coenzyme A deficiency in patients eith PANK2 mutations.. Molecular Genetics And Metabolism.

[9] Dusi S, Valletta L, Haach TB, Tsuchiya Y, Venco P, Pasqualato S, Goffrini P, Tigano M, Demchenko N, Weiland T, Schwarzmayr T, Strom TM, Invernizzi F, Garavaglia B, Gregory A, Sanford L, Hamada J, Bettencourt C, Houldel H, Chiapparini L, Zorzi G, Kurian MA, Nardocci N, Prokisch H, Hayflick S, Gout I, Tiranti V (2014). Exome sequencing reveals mutation in CoA synthase as a cause of neurodegeneration with brain iron accumulation.. Am J Hum Genet.

[10] Campbell GR, Worrall JT, Mahad DJ (2014). The central role of mitochondria in axonal degeneration in multiple sclerosis.. Mult Scler.

[11] Palomo GM, Manfredi G (2014). Exploring new pathways of neurodegeneration in ALS: The role of mitochondria quality control.. Brain Res.

[12] Urrutia PJ, Mena NP, Núñez MT (2014). The interplay between iron accumulation, mitochondrial dysfunction, and inflammation during the execution step of neurodegenerative disorders.. Front Pharmacol.

[13] Kotzbauer  PT,  Truax AC,  Trojanowski JQ, Lee VM (2005). Altered neuronal mitochondrial coenzyme A synthesis in neurodegeneration with brain iron accumulation caused by abnormal processing, stability, and catalytic activity of mutant pantothenate kinase 2.. J Neurosci.

[14] Campanella A, Privitera D, Guaraldo M, Rovelli E, Barzaghi C, Garavaglia B, Santambrogio P, Cozzi A, Levi S (2012). Skin fibroblasts from pantothenate kinase-associated neurodegeneration patients show altered cellular oxidative status and have defective iron-handling properties.. Hum Mol Genet.

[15] Colombelli C,  Aoun M, Tiranti V (2015). Defective lipid metabolism in neurodegeneration with brain iron accumulation (NBIA) syndromes: not only a matter of iron.. J Inherit Metab Dis.

[16] Levi S, Finazzi D (2014). Neurodegeneration with brain iron accumulation: update on pathogenic mechanisms.. Front Pharmacol.

[17] Rinaldi T, Dallabona C, Ferrero I, Frontali L, Bolotin-Fukuhara M (2010). Mitochondrial diseases and the role of the yeast models.. FEMS Yeast Res.

[18] Tenreiro S,  Munder MC,  Alberti S, Outeiro TF (2013). Harnessing the power of yeast to unravel the molecular basis of neurodegeneration.. J Neurochem.

[19] Bleackley MR, MacGillivray RT (2011). Transition metal homeostasis: from yeast to human disease.. BioMetals.

[20] Reinders J, Zahedi RP, Pfanner N, Meisinger C, Sickmann A (2006). Toward the complete yeast mitochondrial proteome: multidimensional separation techniques for mitochondrial proteomics.. J Proteome Res.

[21] Uberbacher EC, Mural RJ (1991). Locating protein-coding regions in human DNA sequences by a multiple sensor-neural network approach.. Proc Natl Acad Sci U S A.

[22] Claros MG, Vincens P (1996). Computational method to predict mitochondrially imported proteins and their targeting sequences.. Eur J Biochem.

[23] Foury F, Cazzalini O (1997). Deletion of the yeast homologue of the human gene associated with Friedreich's ataxia elicits iron accumulation in mitochondria.. FEBS Lett.

[24] Patil Vinay A, Fox Jennifer L, Vishal M, Gohil Dennis R Winge, Miriam L Greenberg (2013). Loss of Cardiolipin Leads to Perturbation of Mitochondrial and Cellular Iron Homeostasis.. J Biol Chem.

[25] Tamarit  J,  Irazusta V,  Moreno-Cermeño A,  Ros J (2006). Colorimetric assay for the quantitation of iron in yeast.. Anal Biochem.

[26] Molik  S,  Lill R,  Mühlenhoff U (2007). Methods for studying iron metabolism in yeast mitochondria.. Methods Cell Biol.

[27] Schilke B, Voisine C, Beinert H, Craig E (1999). Evidence for a conserved system for iron metabolism in the mitochondria of Saccharomyces cerevisiae.. Proc Natl Acad Sci U.S.A..

[28] Mühlenhoff U, Richhardt N, Ristow M, Kispal G, Lill R (2002). The yeast frataxin homolog Yfh1p plays a specific role in the maturation of cellular Fe/S proteins.. Hum Mol Genet.

[29] Wu  M,  Liu H,  Chen W,  Fujimoto Y,  Liu J (2009). Hepatic expression of long-chain acyl-CoA synthetase 3 is upregulated in hyperlipidemic hamsters.. Lipids.

[30] Rana  A,  Seinen E,  Siudeja K,  Muntendam R,  Srinivasan B,  Van der Want JJ,  Hayflick S,  Reijngoud DJ,  Kayser O,  Sibon OC (2010). Pantethine rescues a Drosophila model for pantothenate kinase-associated neurodegeneration.. Proc Natl Acad Sci U S A.

[31] Greenspan P, Mayer EP, Fowler SD (1985). Nile red: a selective fluorescent stain for intracellular lipid droplets.. J Cell Biol.

[32] Kuo YM, Duncan JL, Westaway SK, Yang H, Nune G, Xu EY, Hayflick SJ, Gitschier J (2005). Deficiency of pantothenate kinase 2 (Pank2) in mice leads to retinal degeneration and azoospermia.. Hum Mol Genet.

[33] Yang Y, Wu Z, Kuo YM, Zhou B (2005). Dietary rescue of fumblea Drosophila model for pantothenate-kinase-associated neurodegeneration.. J Inherit Metab Dis.

[34] Nakamura T, Pluskal T, Nakaseko Y, Yanagida M (2012). Impaired coenzyme A synthesis in fission yeast causes defective mitosis, quiescence-exit failure, histone hypoacetylation and fragile DNA.. Open Biol.

[35] Bosveld F, Rana A, van der Wouden PE, Lemstra W, Ritsema M, Kampinga HH, Sibon OC (2008). De novo CoA biosynthesis is required to maintain DNA integrity during development of the Drosophila nervous system.. Hum Mol Genet.

[36] Couplan  E,  Aiyar RS,  Kucharczyk R,  Kabala A,  Ezkurdia N,  Gagneur J,  St Onge RP,  Salin B,  Soubigou F,  Le Cann M,  Steinmetz LM,  di Rago JP, Blondel M (2011). A yeast-based assay identifies drugs active against human mitochondrial disorders.. Proc Natl Acad Sci U S A.

[37] Magni GE, Von Borstel RC (1962). Different rates of spontaneous mutation during mitosis and meiosis in yeast.. Genetics.

[38] Schiestl RH and Gietz RD (1989). High efficiency transformation of intact yeast cells using single stranded nucleic acids as a carrier.. Curr Genet.

[39] Kaiser C, Michaelis S, Mitchell A (1994). Methods in Yeast Genetics..

[40] Ho SN, Hunt HD, Horton RM, Pullen JK, Pease LR (1989). Site-directed mutagenesis by overlap extension using the polymerase chain reaction.. Gene.

[41] Bonneaud N, Ozier-Kalogeropoulos O, Li GY, Labouesse M, Minvielle-Sebastia L, Lacroute F (1991). A family of low and high copy replicative, integrative and single-stranded S. cerevisiae/E. coli shuttle vectors.. Yeast.

[42] Goffrini P, Ercolino T, Panizza E, Giache’ V, Cavone L, Chiarugi A, Dima V, Ferrero I, Mannelli M (2009). Functional study in a yeast model of a novel succinate-dehydrogenase subunit B gene germline missense mutation (C191Y) diagnosed in a patient affected by a glomus tumor.. Hum Mol Genet.

[43] Fontanesi  F,  Soto IC, Barrientos A (2008). Cytochrome c oxidase biogenesis: new levels of regulation.. IUBMB Life.

[44] Barrientos  A, Díaz F (2009). Evaluation of the mitochondrial respiratory chain and oxidative phosphorylation system using polarography and spectrophotometric enzyme assays.. Curr Protoc Hum Genet chapter.

[45] Jarreta D, Orús J, Barrientos A, Miró O, Roig E, Heras M, Moraes CT, Cardellach F, Casademont J (2000). Mitochondrial function in heart muscle from patients with idiopathic dilated cardiomyopathy.. Cardiovasc Res.

[46] Soto IC,  Fontanesi F,  Valledor M,  Horn D,  Singh R,  Barrientos A (2009). Synthesis of cytochrome c oxidase subunit 1 is translationally downregulated in the absence of functional F1F0-ATP synthase.. Biochim Biophys Acta.

[47] Bradford MM (1976). A rapid and sensitive method for the quantitation of microgram quantities of proteins utilizing the principle of protein dye binding.. Anal Biochem.

[48] Glick BS, Pon LA (1995). Isolation of highly purified mitochondria from Saccharomyces cerevisiae.. Methods Enzymol.

[49] Diekert K, De Kroon AI, Kispal G, Lill R (2001). Isolation and subfractionation of mitochondria from the yeast Saccharomyces cerevisiae.. Methods Cell Biol.

[50] Trott A and Morano KA (2004). SYM1 is the stress-induced Saccharomyces cerevisiae ortholog of the mammalian kidney disease gene Mpv17 and is required for ethanol metabolism and tolerance during heat shock.. Eukaryot Cell.

[51] Almeida T,  Marques M,  Mojzita D,  Amorim MA,  Silva RD,  Almeida  B,  Rodrigues P,  Ludovico P,  Hohmann S,  Moradas-Ferreira P,  Côrte-Real M, Costa V (2008). Isc1p plays a key role in hydrogen peroxide resistance and chronological lifespan through modulation of iron levels and apoptosis.. Mol Biol Cell.

[52] Kimura K, Yamaola M, Kamisaka Y (2004). Rapid estimation of lipids in oleaginous fungi and yeasts using Nile red fluorescence.. J Microbiol Methods.

[53] Sitepu IR, Ignatia L, Franz AK, Wong DM, Faulina SA, Tsui M, Kanti A, Boundy-Mills K (2012). An improved high-throughput Nile red fluorescence assay for estimating intracellular lipids in a variety of yeast species.. J Microbiol Methods.

